# Slower tempo makes worse performance? The effect of musical tempo on cognitive processing speed

**DOI:** 10.3389/fpsyg.2023.998460

**Published:** 2023-02-24

**Authors:** Hung-Ming Lin, Su-Hui Kuo, Thao Phuong Mai

**Affiliations:** ^1^Department of Business Administration, Minghsin University of Science and Technology, Xinfeng, Hsinchu, Taiwan; ^2^Department of Business Administration, National Changhua University of Education, Changhua City, Taiwan

**Keywords:** musical tempo, cognitive processing speed, task performance, arousal, accuracy

## Abstract

The effects of musical tempo on cognitive processing speed were investigated, and the mediating effect of arousal was empirically tested. In an experiment, participants were divided into fast tempo, slow tempo, and no-music groups and completed three cognitive processing speed tests measuring motor speed, visuospatial processing speed, and linguistic processing speed. The results indicated a significant effect of musical tempo on processing speed and task performance in all three tasks. The slow-tempo group exhibited slower processing speed and worse performance than the no-music group in all three tasks. The fast tempo group displayed no significant difference in processing speed or performance compared with the no-music group. In the linguistic processing task, those who listened to slow-tempo music had better accuracy than those in the other conditions. Arousal did not mediate the relationship between musical tempo and cognitive processing speed.

## 1. Introduction

Music is widely used to stimulate human behaviors and to influence information processing. With a long history in human civilization, music has played a prominent role in human mental life regardless of geography or race (Morley, 2013). In neuroscience, Dillman Carpentier and Potter (2007) reported that higher frequency concerts elevate the skin conductance response; rock music produces a higher skin conductance response compared with classical music because of its higher frequency.

In education, studies have indicated that when hearing music, the hemispheres of the brain work together to process the melody and analyze the structural components of the music while the limbic system activates an emotional response, and the bilateral cooperation strengthens the connection between synapses and exercises the brain cells, which in turn assists children with learning and emotion processing (Foran, 2009; Schwartz et al., 2017). Hallam and Price (1998) investigated a class of children with emotional and behavioral difficulties and reported that background music significantly improved their rule-following behavior and performances in math tasks. Sacks (2007) concluded that music can also improve memory skills because sequences of knowledge are activated when hearing an associated piece of music. These studies have demonstrated improvement in the abilities of students when background music is playing.

In the workplace, music has also been widely used to boost performance and motivation (Styhre, 2013; Landay and Harms, 2019). For example, Geethanjali et al. (2016) measured the task performance and associated physiological changes of participants listening to music (Indian classical instrumental or Indo jazz). Changes in the mean pulse rate were significantly lower with Indo jazz, and both genres induced positive effects and enhanced task performance. According to these findings, music can play a critical role in the workplace.

However, the use of background music to aid productivity has been also used in an unstandardized manner causing reverse effects, such as by distracting people and thus slowing working processes and decreasing overall work efficiency (Avila et al., 2012), thus, understanding of music’s effect on productivity seems incomplete. Recent studies of the influence of background music on student task performance have reported mixed and inconsistent results. Whereas many researchers have indicated significant improvement in student performance in math (Graziano et al., 1999) and comprehension tasks (Anderson and Fuller, 2010) as well as concentration level (Smith and Morris, 1977) and memory (Salamé and Baddeley, 1989; Irish et al., 2006), many others have indicated that music also causes distraction (Smith and Morris, 1977; Avila et al., 2012; Doyle and Furnham, 2012).

Although numerous studies have examined how music influences learning activities, researchers have tended to focus on emotional and arousal factors and have paid less attention to speed. For example, Kallinen (2002). indicated that music can actually mask distractions when reading; university students read faster and remembered more content when the background music in a cafeteria consisted of fast-tempo classical music compared with conditions in which music was slower or absent whatever the effect of musical tempo on learning activities, its effect on task performance is less clear. Studies of the effect of background music on task performance have focused on reading, writing, and physical tasks. With the development of the Internet, the effects of background music on motor, visuospatial processing, and linguistic processing speed and the associations with learning or task performance have practical implications for education. Furthermore, time perception plays a key role in task performance. Time management and task completion speed are equally important in achieving efficiency and results in a limited time (Macan et al., 1990). Therefore, the aspect of time requires additional attention for a more complete understanding of how music affects education. The current study investigates how musical tempo affects the information processing speed of students as well as the mechanism behind its role.

## 2. Literature review

### 2.1. Effect of background music on productivity

The effect of background music on learning has been a matter of debate among researchers due to inconsistent findings. Nevertheless, that music has generally positive effects on learning is undeniable. Music affects human behavior and psychological or physiological phenomena. For example, scholars have addressed that fast-paced music gives the impression of being happy and pleasant, whereas slow-paced music prompts the feeling of quiet, sadness, and solemnity (Gundlach, 1932; Hevner, 1937; Rigg, 1940; Watson, 1942; Scherer and Oshinsky, 1977). Dillman Carpentier and Potter (2007) explored the influence of music on physiological arousal and discussed it in terms of rhythm and type. Their results indicated that a fast rhythm makes the skin computer reflect the slower rhythm of music higher, and the skin conductance measurement index suggested that faster rhythmic music triggers a higher degree of arousal than slow-paced music.

At the behavioral level, some evidence has indicated that the presence of background music increases student concentration, attitude, and motivation in classes (Hallam and Price, 2003; Bloor, 2009). For example, Lehmann and Seufert (2017) investigated the influence of background music on learning. They reported that both the Mozart effect and the arousal–mood-hypothesis indicate that background music can potentially promote learning outcomes. Whereas the Mozart effect assumes a direct influence of background music on cognitive ability, the arousal–mood-hypothesis assumes a mediation effect over arousal and mood. Lehmann et al. (2019) further explored the influence of background music on learners with varying degrees of extraversion; the results suggested that seductive details in general negatively affect learning and cognitive load. However, background music as a seductive detail may also influence the learner’s arousal, whose optimal level depends on their level of extraversion. Earlier studies have indicated that music may stimulate the production of endorphins in the brain’s limbic system, which directly affects the physiological parameters of blood pressure, body temperature, and pulse rate and helps slow body metabolism and reduce enzyme and hormone production in students (Savan, 1999).

In a study on music preference exploring the effect of background music on worker concentration, the influence of background music on listener attention depended on fondness for the music more than the type of music. The authors suggested not selecting background music that workers strongly like or dislike to avoid negatively affecting worker concentration (Huang and Shih, 2011). The effect of music on cognitive performance has received considerable attention among researchers. Music playing in the background has been proved to enhance memory, creativity, and cognitive processing (Salamé and Baddeley, 1989; Hetland, 2000; Rainey and Larsen, 2002; Jaušovec et al., 2006; Dosseville et al., 2012; Fassbender et al., 2012; Legutko and Trissler, 2012; Ritter and Ferguson, 2017).

In contrast to the findings of these studies, other studies have suggested that listening to background music distracts cognitive processing among students for various reasons (Furnham et al., 1999; Jing et al., 2012). Background music that includes vocals and has a high level of familiarity is considered to cause the most distraction in task performance (Avila et al., 2012). Music type is also a key indicator of the distraction effect on thinking processes. Music that is aggressive and has a higher intensity, such as rock or hip hop, tends to negatively affect the concentration of students, while calming and relaxing music such as classical and instrumental music tends to have more positive effects on cognitive performance (Hallam and Price, 2003). However, Thompson et al. (2012) claimed that fast instrumental music at high volume also disrupted the cognitive processing of students; thus, inappropriate music choice provides no benefits and may negatively affect learning. However, evidence for the effects of background music on concurrent and subsequent cognitive performance appears inconsistent.

### 2.2. Musical tempo and processing speed

In music, tempo refers the speed or pace of a piece of music and is usually measured in beats per minute (bpm). Research on musical tempo has generally compared the effect that fast and slow-tempo music produces on human activity and behavior. In fact, Studies have reported the effects of musical tempo on behavior and productivity in a variety of contexts such as in eating behavior (Spence, 2011; Spence and Piqueras-Fiszman, 2014; Spence et al., 2019), the pace of behavior (Kuribayashi and Nittono, 2015), and emotional autonomous response (Orini et al., 2010). For example, Mathiesen et al. (2020) investigated the extent to which specific musical properties, namely tempo and articulation, influence eating duration, and they found increase in eating duration while listening to music, compared to silence, and this finding could be explained as a result of music distracting attention when people are in the eating activity. In neuroscience, Orini et al. (2010) explored continuous assessment of the emotional autonomous response to music. They found that compared with the rest state, during listening to pleasing music, various stimuli caused significant changes, and the heart and breathing rate were higher, and compared with the rest state.

Regarding cognitive performance, musical tempo may have a comparable effect on processing speed. Researchers have reported that faster tempo music tends to enhance memory (Chie and Karthigeyan, 2009), increase accuracy in decision-making tasks (Day et al., 2009), and, in general, produce better performance (Mayfield and Moss, 1989). Day et al. explored the effects of musical tempo and task difficulty on multi-attribute decision-making by using an eye-tracking approach. They concluded that in the same decision-making time, participants made decisions more accurately with the presentation of faster music. In addition, faster music improved the accuracy of challenging but not that of easier decision-making tasks. In addition, Geethanjali et al. (2016) explored the enhancement of task performance aided by music and reported that task performance and associated physiological changes were observed in participants who listened to music (Indian classical instruments or Indo jazz); changes in the mean pulse rate were significantly lower with Indo jazz. Indian classical instruments and Indo jazz induced positive effects and enhanced task performance. Accordingly, music can play a critical role in the workplace. In summary, musical tempo influences cognitive processing speed and task performance.

Processing speed is defined as the amount of time required to perform a mental task or the amount of work that can be completed within a certain period (Braaten and Willoughby, 2014). Sweet (2011) called this information processing speed (IPS), which measures the efficiency of cognitive functions and is expressed as reaction time, meaning the time between receiving and responding to a stimulus. Processing speed depends on genetic factors but can change over a lifetime due to environmental factors (Edwards et al., 2005; Nouchi et al., 2012; Sandroff and Motl, 2012). Numerous studies have indicated that music listening and training can positively influence cognitive functions including processing speed (Walker et al., 2012). Musical tempo can have a dramatic influence on the speed of cognitive processing. The present study focused on motor speed, visuospatial processing speed, and linguistic processing speed.

Musical tempo should have a positive effect on processing speed. An experiment on workplace productivity by Mayfield and Moss (1989) compared heartbeat music and fast-paced rock music; the results indicated that the slow pace of heartbeat music slowed workers, whereas rock music resulted in better and faster performance even though it caused distraction and a stressful emotion state. This indicates that music with a faster tempo tends to increase the speed of actions. This is likely to hold true for cognitive processing speed; therefore, we propose the following hypothesis:

*H1*: Background music with a fast (slow) tempo significantly increases (decreases) cognitive processing speed in comparison to slow (fast) tempo music and no-music conditions.

### 2.3. Arousal as a mediator of the relationship between musical tempo and processing speed

Arousal is the physiological and psychological state of being awake. The effects of physiological arousal on cognition cause individuals to be active, attentive, or excited. Physiological arousal refers to features of arousal involving physiological responses, such as increases in blood pressure and rate of exhalation and narrowing of the gastrointestinal system (Zhou and Siu, 2015). Dillman Carpentier and Potter (2007) explored the influence of music on physiological arousal. They compared fast-paced rock music and slow-paced classical music and reported that higher frequency produced a higher skin conductance response; rock music produced a higher skin conductance response compared with classical music because of its higher frequency. In the earlier study by Holbrook and Anand (1990), the effects of tempo and situational arousal on the listener’s perceptual and affective responses to music were explored, and the results were suggestive of a psychophysical relationship between musical tempo and perceived activity. Although musical tempo affects physiological responses, the Yerkes–Dodson law suggests that an optimal level of arousal for performance exists, and too little or too much arousal can harm cognitive performance (Yerkes and Dodson, 1908).

The effect of music on arousal level and mood is widely considered the principal mechanism behind these benefits (Hallam et al., 2002). Kotsopoulou and Hallam (2010) claimed that the superior performance of students in memory tasks was mediated by arousal and mood rather than the result of a direct effect on cognition. In a recent experiment on the effect of specific types of music on divergent thinking, Ritter and Ferguson (2017) observed that creativity was higher for participants who listened to “happy music” (arousing classical music with a positive mood) compared with that of participants who performed the creative task in silence.

Hu et al. (2019) tested the effect of different types of background music on the reading comprehension of postgraduate students and reported that the emotional changing function of music is more prominent when it is listened to with focused attention before the test. With respect to the mechanism behind these effects, as discussed, arousal and mood level have been assumed to be the main mediators of the relationship between musical tempo and cognitive performance, but little empirical evidence is available to support this theory.

Throughout the history of, most researchers who have studied the effect of music on cognitive performance have believed that the principal mechanism is due to the change in emotion and arousal level that faster musical tempos can produce. This theory started with the famous Mozart effect, which explained that musical tempo can trigger emotional and arousal states which, in turn, enhance motivation and alertness, resulting in more favorable performance in cognitive tasks. Studies focusing on temporal factors have also apply this theory and indicated that musical tempo links directly with arousal changes and, as a result, affects the speed of activities. For instance, in a study on eating speed, Roballey et al. (1985) claimed that the arousal effect was the main cause of an increase in the average number of bites per minute when listening to faster music. In study of Mayfield and Moss (1989), he also explained that rock music made the participants feel more hurried, and they worked faster as a result. Therefore, arousal should mediate the relationship between musical tempo and processing speed.

*H2*: Arousal mediates the effect of musical tempo on processing speed.

Concluding from the hypotheses presented above, and we hypothesized that the musical tempo has positive influences processing speed, and psychological arousal mediates the effect of musical tempo on processing speed.

## 3. Methodology

### 3.1. Experimental design and procedure

An experimental method using a computer-based test was adopted to measure the cognitive processing speed of students while fast- or slow-paced music was played in the background. Overall, 29 male (55.8%) and 23 female (44.2%) Vietnamese undergraduate students (*M* = 23.7 years, *SD* = 4.94 years) were recruited, and their background related management, engineering, humanities, design, technology etc. One outlier was excluded from the dataset, and the remaining 51 samples were analyzed. With 0.05 for α, 0.80 for statistical power level, and 51 for sample sizes, the calculator G^*^power suggests effect size is 0.611 (Cohen, 1988).

Participants were randomly divided into three groups for the processing speed test. The three conditions were fast tempo (FT), slow tempo (ST), and no music (NM) as the control group. The FT and ST groups listened to music while doing the test, and the NM group did the test without background music. Refer to experimental design of Chie and Karthigeyan (2009), the music was played when the participants started reading the introduction to the experiment and the consent form; thus, it was playing for approximately 1–2 min prior to starting the test.

Before the test, the participants were asked demographic questions, such as on their age, gender, and whether they played an instrument, followed by two seven-point Likert-scale questions asking how often they listen to music (Question 1: “I often listen to music”; Question 2: “I often listen to music while studying”; 1 = never to 7 = very often). They then completed three cognitive tasks designed to measure processing speed. After finishing the test, the participants were asked to answer a questionnaire that included items designed to explore the possible mechanism behind the effect of musical tempo on processing speed.

### 3.2. Manipulation of musical tempo

Musical tempo was manipulated. The Mozart Sonata for Two Pianos in D major, K. 448 (Mozart, 1985) was selected as auditory stimulus because it was suggested by the British Epilepsy Organization to have the Mozart effect. The Sonata has three movements: Allegro (fast tempo), Andante (fairly slow tempo; at a walking pace), and Molto Allegro (fast tempo; slightly faster than Allegro). The second movement (Andante) was selected, and software was used to change the tempo of the piece to create an FT version (138 bpm) and an ST version (66 bpm).

### 3.3. Measurement of dependent variable

Three computer-based reaction time tests were used to measure different types of processing speed. The choice reaction time (CRT) test measured motor speed, the visual search (*VS*) test measured visuospatial processing speed, and the letter name matching (LNM) test measured linguistic processing speed. These are standardized tests that are widely used to assess information processing speed (IPS).

**Figure 1:**
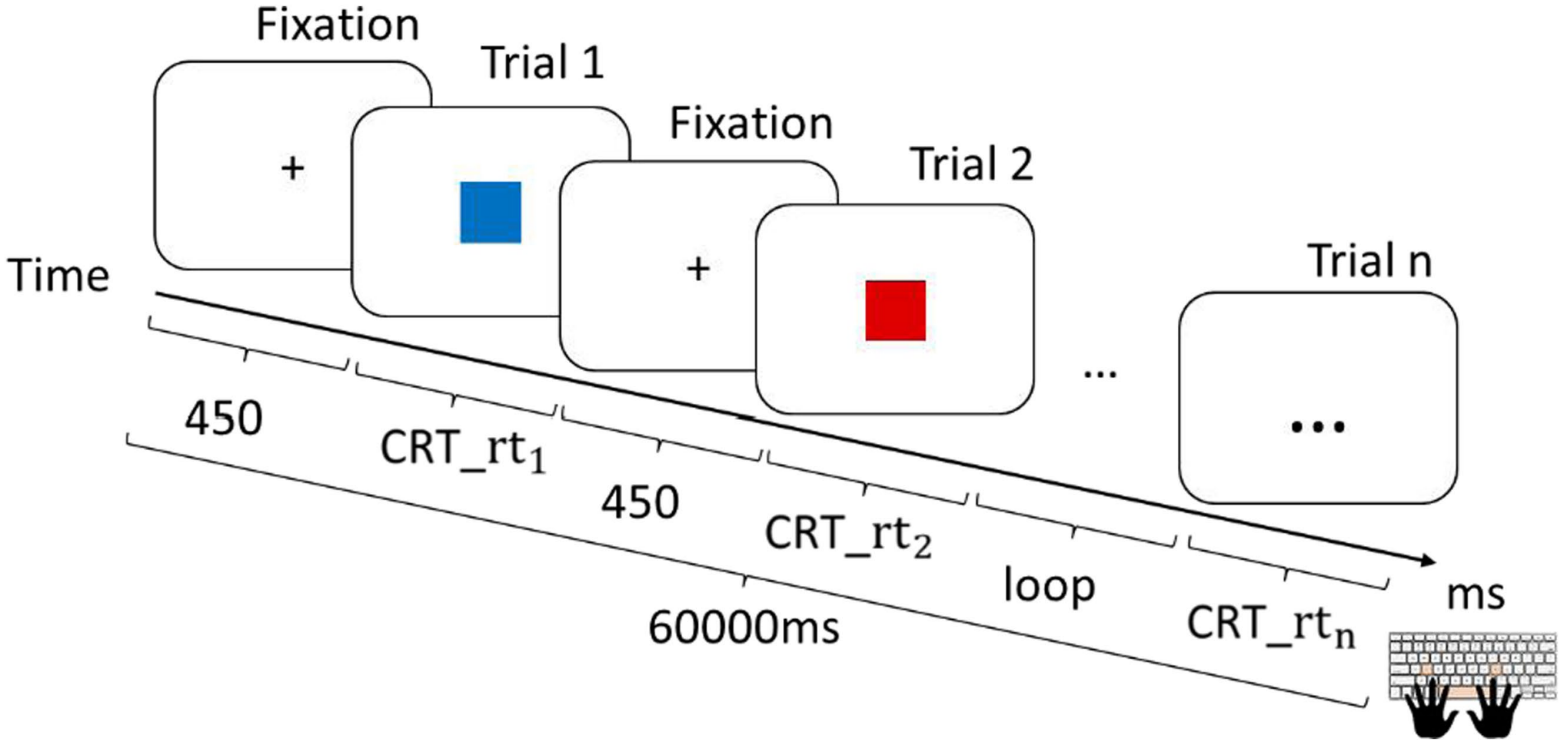
Trial sequence of the CRT task. rt, reaction time.

Motor speed (Ruff and Parker, 1993), or “psychomotor speed,” is the subject’s speed of performing a rapid motor response to a sudden signal. It includes components such as simple reaction time, CRT, and speed of movement (Kauranen, 1999). The CRT test is one of the three tasks of the Computerized Test of Information Processing (Tombaugh et al., 2010). In the task, participants see two stimuli, each of which is associated with a particular response (e.g., right button for red squares and left button for blue squares). Participants use a keyboard to choose the appropriate response as quickly as possible (Figure 1).

**Figure 2:**
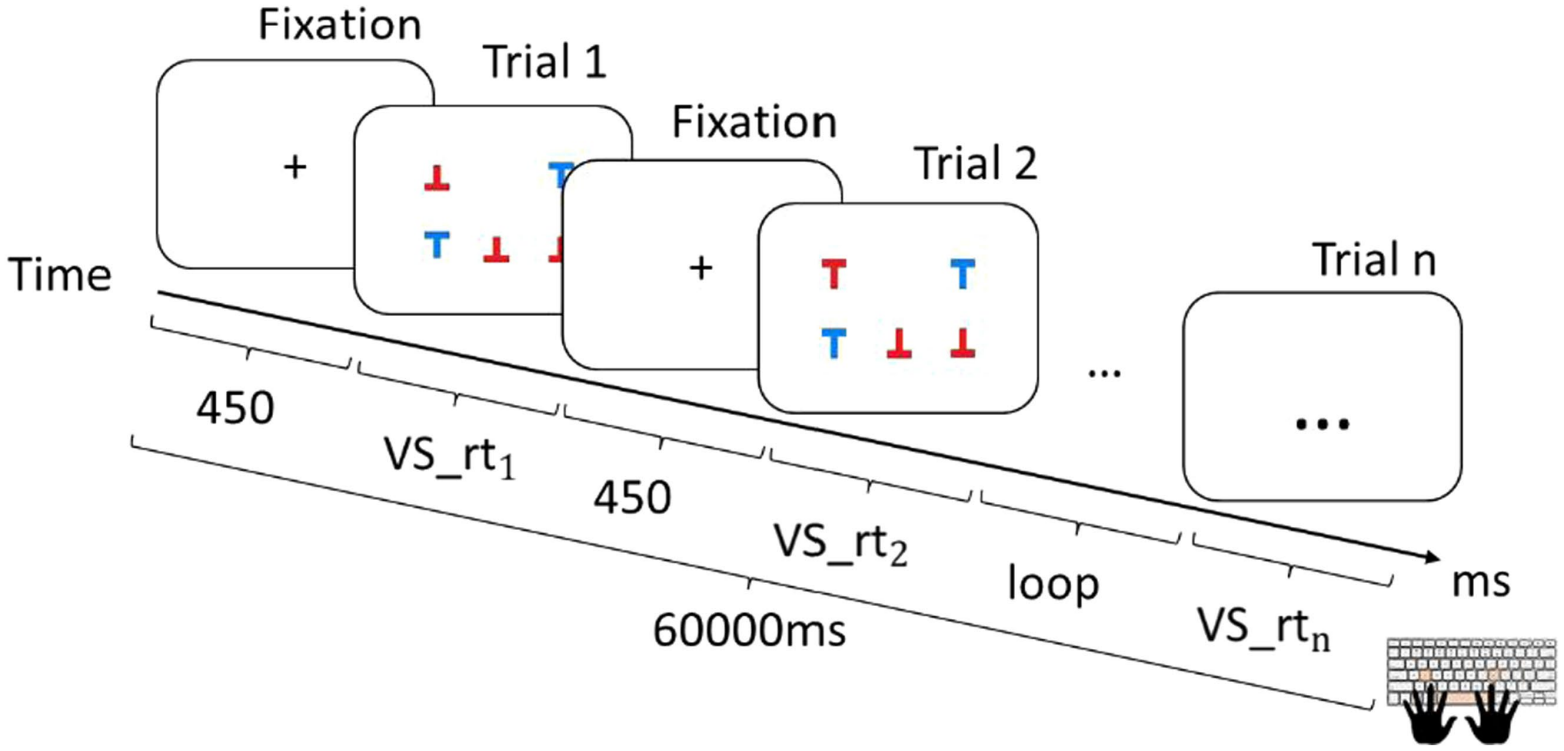
Trial sequence of the *VS* task. rt, reaction time.

Visuospatial processing speed is the speed at which individuals “perceive, analyze, synthesize, manipulate and transform visual patterns and images” (Dehn, 2011). Visuospatial speed is central to efficiency in distance perception and spatial navigation (Bradford and Atri, 2014). In the *VS* test (Wolfe, 2018), participants must respond to the letter “T,” but only if it is in a regular upright position and only if it is red. The participants decide if the letter appears upright in red and respond using left and right buttons as quickly as possible (Figure 2).

**Figure 3:**
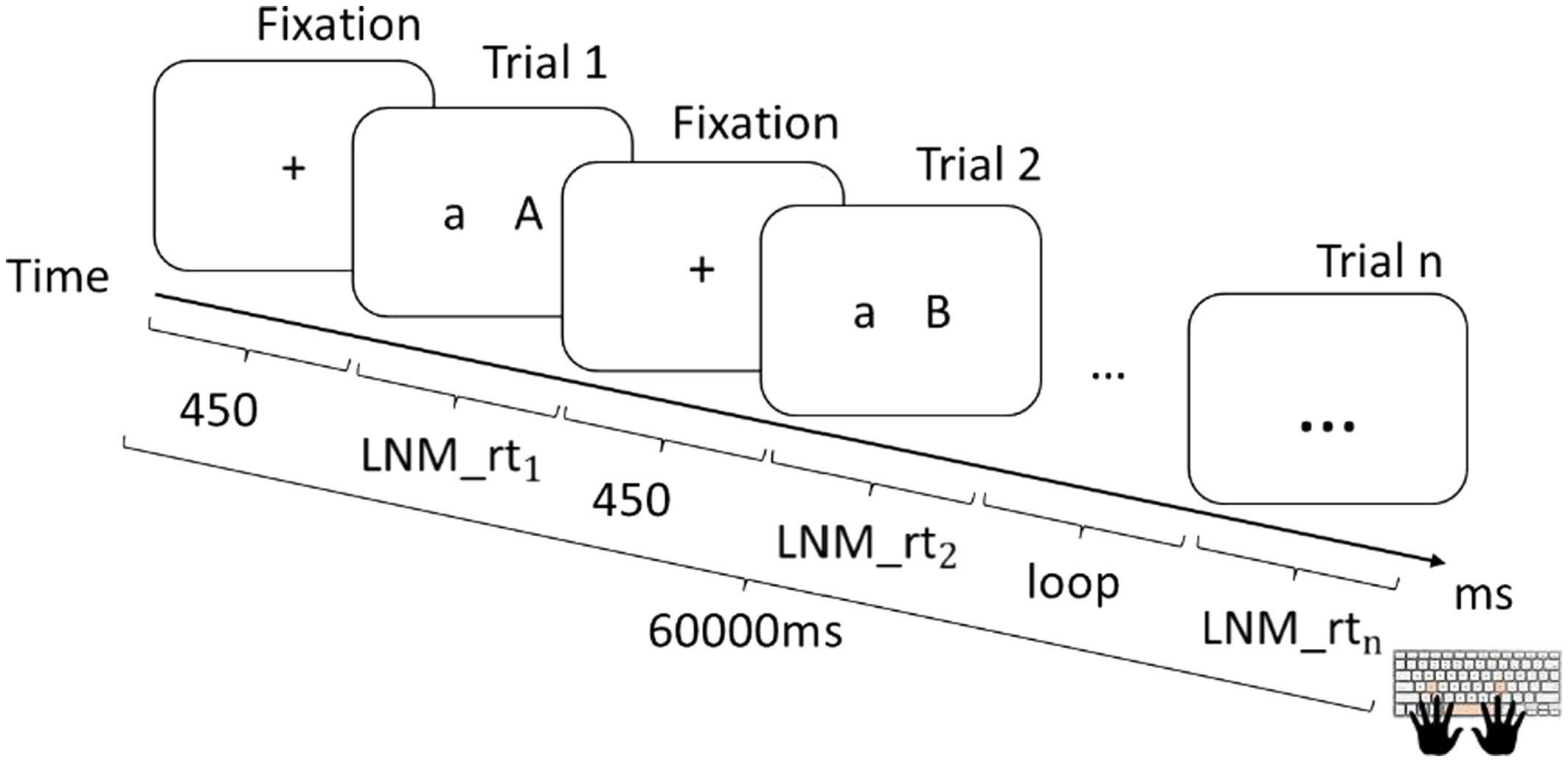
Trial sequence of the LNMtask. rt, reaction time.

For linguistic processing speed, the (LNM) test, which is a letter-matching task developed by Posner and Mitchell (1967), was adopted. Participants are presented with two letters on the screen and must decide whether they have the same or different names when one is upper case and the other is lower case, as quickly as possible. This task is based on the classical Posner task and is widely used to assess semantic processing speed (Figure 3).

These tasks enabled us to record reaction time or response time—the amount of time a person requires to perceive, process, and react to a stimulus. Each task had 100 trials. Between each trial, a fixation-dot appeared for participants to focus on the middle of the screen, which lasted 450 ms. The time limit for each task was 1 min (60,000 ms); thus, the participants would not be able to finish all the trials with correct answers. The highest possible score for each task was 100 points. Before each task, instructions were given, and each participant had two practice trials to ensure that they understood the task.

### 3.4. Follow-up questionnaire

**Figure 4:**
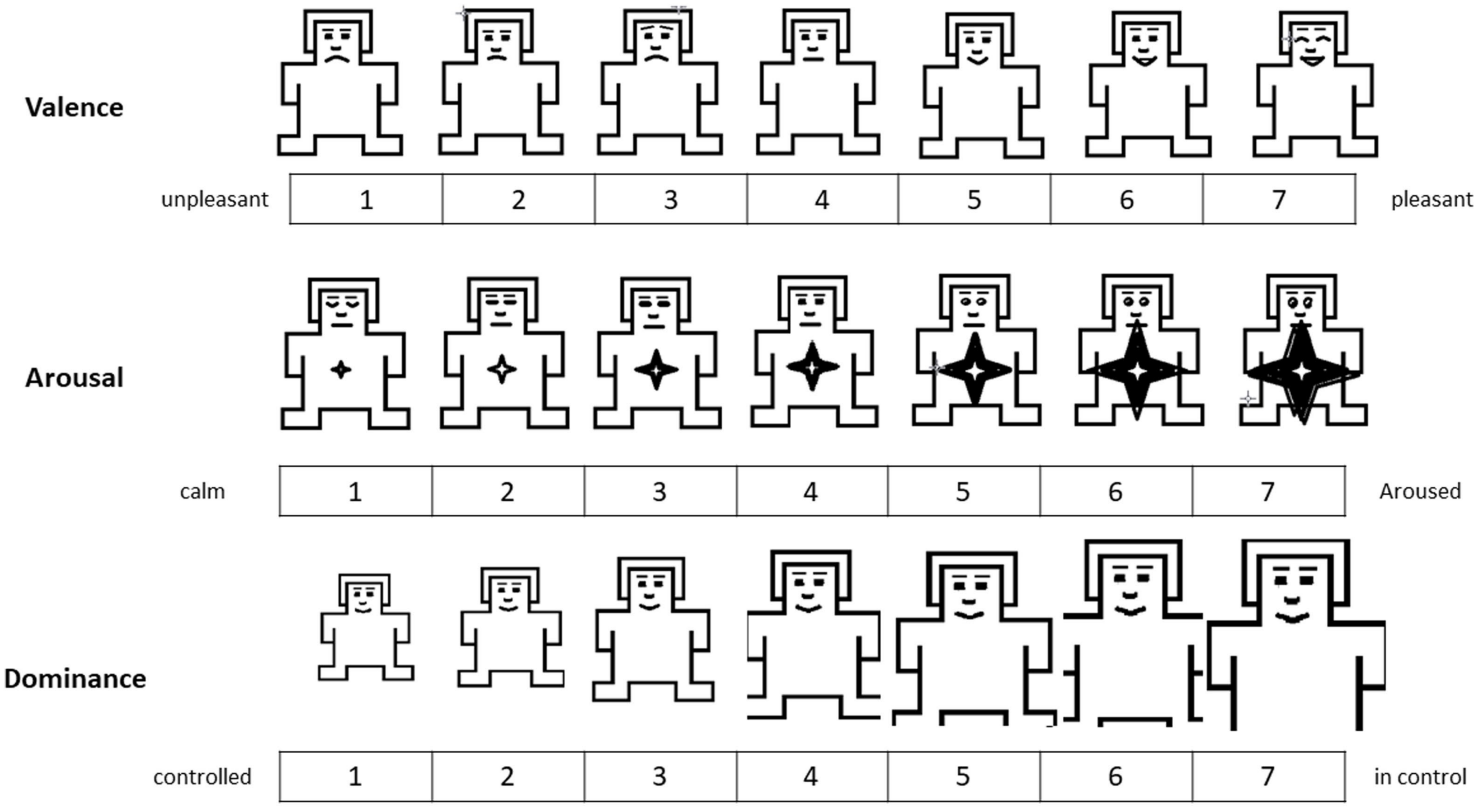
Emotion assessment using the SAM.

To gain further understanding of whether arousal and mood constitute the underlying mechanism of the relationship between musical tempo and processing speed, a follow-up questionnaire was provided after the participants had completed the tasks. The Self-Assessment Manikin (SAM), a popular picture-oriented questionnaire designed to measure three psychological features of emotion, namely valence, arousal, and dominance (Bradley and Lang, 1994), was employed. This cross-cultural pictorial style of self-report assessment was selected because it is reliable and has been widely used to measure emotional states (Smith and Morris, 1977). Use of questionnaire eliminated most of the potential risks of false translation and misunderstanding. Figure 4 presents the SAM version used in the present study. The three affective dimensions were measured using seven-point scales: valence (1 = “unpleasant” to 7 = “pleasant”), arousal (1 = “calm” to 7 = “aroused”), and dominance (1 = “controlled” to 7 = “in control”).

## 4. Results

Fifty-one valid samples participate in the study. Their average age was 23.7 years old (SD = 4.94). Among these participants, 25 said that they play instruments (48.1%), 27 does not play instruments (51.9%). For the question “I often listen to music,” 63.5% of participants responded “often” to “extremely often,” 28.8% responded “rarely” to “never,” and 7.7% had neutral answer. For the question “I often listen to music while studying,” 55.8% of participants answered “often” to “extremely often,” 34.6% answered “rarely” to “never” and 9.6% had neutral answer. It indicates that the majority of participants have the habit of listening to music in daily life and while studying.

### 4.1. Influence of musical tempo on CRT

The CRT task tested motor processing speed. A significant main effect was observed for processing speed in the three conditions; *F*(2,49) = 10.285, *p* < 0.05, *η^2^* = 0.580. The main effect of score was *F*(2,49) = 35.651, *p* < 0.05, *η^2^* = 0.543. The accuracy rates under the three conditions did not significantly differ; *F*(2,49) = 0.244, *p* > 0.05, which indicates that speed differences were the main driver of the differences in performance in this task.

*Post hoc* comparisons indicated that the speed of the participants in the ST condition (*M* = 916.5, *SD* = 447.2) was considerably lower than that of those in the FT (*M* = 553.6, *SD* = 85.4) and NM (*M* = 564.2, *SD* = 74.4) conditions. In addition, scores under the ST condition were much lower (*M* = 25.9, *SD* = 7.9) than under the FT (*M* = 45.5, *SD* = 5.9) and NM (*M* = 45.2, *SD* = 10.4) conditions. However, no significant difference in performance or speed was observed between the FT and NM groups, indicating that fast-tempo music did not improve the speed or performance of the participants (Table 1).

**Table 1:** Mean and standard deviation of variables in choice reaction time (CRT) task.

	Slow tempo (*n* = 18)		Fast tempo (*n* = 20)		No-music (*n* = 13)		*F* (2,49)	*Post hoc*
	*M*	*SD*	*M*	*SD*	*M*	*SD*		
CRT reaction time (ms)	916.5	447.2	553.6	85.4	558.2	72.6	10.420^*^	ST > FT
								ST > NM
CRT score	25.9	7.89	45.4	5.94	45.1	10.4	35.651^*^	ST < FT
								ST < NM
CRT accuracy rate (%)	95.8	4.21	95.7	4.10	96.6	3.36	0.244	

M and SD represent mean and standard deviation, respectively, ^*^p < 0.05.

### 4.2. Influence of musical tempo on *VS*

Results for the *VS* task testing visuospatial processing speed indicated a significant main effect for background music condition on reaction time; *F*(2,49) = 18.909, *p* < 0.05, *η^2^* = 0.436. In addition, the main effect on test score was *F*(2,49) = 10.465, *p* < 0.05, *η^2^* = 0.299. The results of *post hoc* testing exhibited a trend similar to that of the CRT test. The ST group displayed markedly more reaction time (*M* = 1,025.7, *SD* = 209) than did the FT (*M* = 869.9, *SD* = 186.3) and NM (*M* = 837.5, *SD* = 102) groups. In addition, the ST condition had a significantly worse performance (*M* = 18.3, *SD* = 6.28) than the FT (*M* = 27.04, *SD* = 7.83) and NM (*M* = 28.38, *SD* = 3.64) conditions did.

The FT and NM conditions did not exhibit a significant difference in any criteria. Regarding accuracy, no significant difference was observed among the three conditions; *F*(2,49) = 1.807, *p* > 0.05. This indicates that slow-tempo music may decrease the speed of visuospatial processing, but a fast tempo does not increase this processing speed compared with the quiet condition (Table 2).

**Table 2:** Mean and standard deviation of variables in visual search (*VS*) task.

	Slow tempo (*n* = 18)		Fast tempo (*n* = 20)		No-music (*n* = 13)		*F* (2,49)	*Post hoc*
	M	SD	M	SD	M	SD		
*VS* reaction time (ms)	1025.7	209	869.9	186.3	837.5	102	16.805^*^	ST > FT
								ST > NM
*VS* score	18.3	6.28	27.04	7.83	28.38	3.64	12.230^*^	ST < FT
								ST < NM
*VS* accuracy rate (%)	97.07	4.78	91.77	10.3	94.4	3.02	2.562	

M and SD represent mean and standard deviation, respectively, ^*^p < 0.05.

### 4.3. Influence of musical tempo on LNM

Regarding the LNM task, which is more difficult and more likely to have incorrect answers, the analysis revealed slightly different results. The main effect of musical tempo on LMN reaction time was significant; *F*(2,49) = 22.3, *p* < 0.05, *η^2^* = 0.476. The result of the *post hoc* test indicated that the speed of the participants under the ST condition (*M* = 1,125, *SD* = 304.3) was substantially lower than that of those under the FT (*M* = 717.4, *SD* = 108) and NM (*M* = 788.7, *SD* = 96.7) conditions.

The final task score also revealed a significant main effect difference in the three conditions; *F*(2,49) = 14.661, *p* < 0.05, *η^2^* = 0.374. The score of the ST group (*M* = 16.8, *SD* = 5.33) was notably lower than that of the FT (*M* = 25.6, *SD* = 5.2) and NM (*M* = 22.7, *SD* = 4.44) groups.

The accuracy rates for the three conditions were significantly different; *F*(2,49) = 5.338, *p* < 0.05, *η^2^* = 0.179. The *post hoc* test results indicated that the accuracy of the ST participants (*M* = 93.4, *SD* = 5.05) was much higher than that of the NM participants (*M* = 87.1, *SD* = 4.9) and somewhat higher than that of the FT participants (*M* = 90, *SD* = 5.97). This task measured linguistic processing, which is more difficult; therefore, in tasks that are more complex, slower music may be beneficial for accuracy (Table 3).

**Table 3:** Mean and standard deviation of variables in letter name matching (LNM) task.

	Slow tempo (*n* = 18)		Fast tempo (*n* = 20)		No-music (*n* = 13)		*F* (2,49)	*Post hoc*
	M	SD	M	SD	M	SD		
LNM reaction time (ms)	1,125	3.4.3	722.6	101	788.7	96.7	22.128^*^	ST > FT
								ST > NM
LNM score	16.8	5.33	25.6	5.19	22.6	4.44	14.661^*^	ST < FT
								ST < NM
LNM accuracy rate (%)	93.4	5.05	89.9	5.97	87.1	4.9	5.338^*^	ST > NM

M and SD represent mean and standard deviation, respectively, ^*^p < 0.05.

Generally, the results of the three cognitive tasks suggest that tempo influenced the processing speed of the participants. Specifically, the processing speed of the participants under the ST condition tended to be slower than that of the participants under the NM condition, with worse performances. Fast music did not significantly increase processing speed and, as a result, did not provide benefits for overall performance in any of the tasks.

### 4.4. Mediation analysis

Finally, to identify potential mediators of the relationship, we use the mediation analysis method of the Process macro (v3.5) model 4 developed by Hayes (2017) in SPSS. The current study investigated whether emotion factors mediated the relationship between musical tempo and processing speed, and multiple mediation analysis was conducted with three variables: valence, arousal, and dominance.

**Figure 5:**
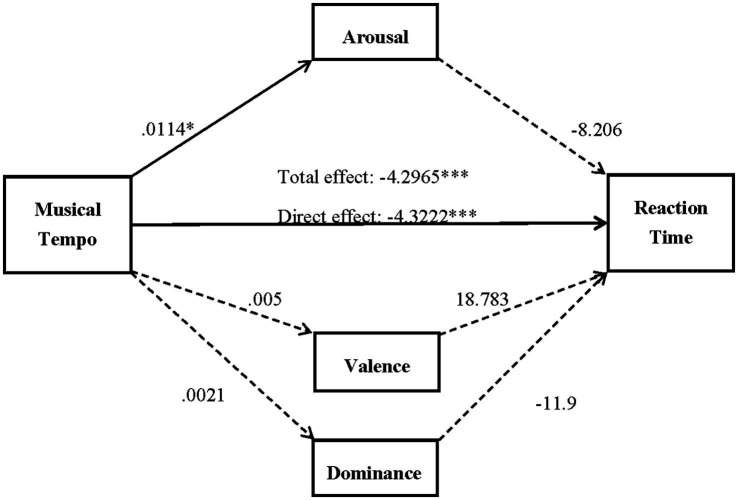
Unstandardized coefficients of the mediation model. ^*^p < 0.05, ^**^p < 0.01, and ^***^p < 0.001.

The direct effect of musical tempo on arousal was positive and significant, indicating that musical tempo positively influenced arousal. The direct effects of musical tempo on valence and dominance were positive but nonsignificant, revealing that musical tempo did not influence valence or dominance (Figure 5).

The direct effect of musical tempo on reaction time was negative and significant, indicating that the participants in the fast music group were more likely to have a shorter reaction time (i.e., faster processing speed). The direct effect of arousal, valence, and dominance on reaction time was not significant, revealing that arousal, valence, and dominance did not influence reaction time.

Regarding the indirect effect, the nonparametric bootstrapping analysis with 95% confidence intervals indicated that the indirect effect of musical tempo on processing speed through arousal, valence, and dominance was not significant. Therefore, arousal, valence, and dominance did not mediate the relationship between musical tempo and processing speed (Tables 1, 4).

**Table 4:** Results of mediation analysis.

Direct effect	Mediator									Dependent variables		
	Arousal			Valence			Dominance			Reaction time		
	$R^{2}\;$= 0.1192			$R^{2}$ = 0.0311			$R^{2}$ = 0.0076			$R^{2}$ = 0.725		
	B	SE	t	B	SE	t	B	SE	t	B	SE	t
Arousal	-	-	-							−8.21	15.4	−0.5337
Valence				-	-	-				18.78	17.0	1.099
Dominance							-	-	-	−11.9	20.1	0.5906
Musical Tempo	0.011	0.005	2.2^*^	0.005	0.005	1.07	0.002	0.004	0.524	−4.32	0.501	−8.634^***^
Indirect effect										Effect	SE	CI
Through arousal										−0.093	0.217	[−0.561, 0.339]
Through valence										0.094	0.13	[−0.155, 0.371]
Through dominance										0.025	0.087	[−0.141, 0.229]

^*^p < 0.05, ^**^p < 0.01, ^***^p < 0.001.

## 5. Conclusion

The present study is among the first to reveal the effect of musical tempo on cognitive processing speed, which is crucial in cognitive performance. The primary objective of the study was to determine whether music tempo has an effect on processing speed and, hence, cognitive performance. We hypothesized that fast-tempo music may increase cognitive speed and improve cognitive performance, whereas slow music may decrease the speed of cognitive processing and, in turn, worsen cognitive performance (H1).

The results of the experiment with three cognitive tasks indicated that fast music does not significantly increase cognitive speed compared with the quiet condition. Cognitive performance, therefore, is not significantly better when listening to fast music. This result was in contrast to those of some previous studies on driving speed (Brodsky, 2001), eating speed (Roballey et al., 1985), physical speed (Mayfield and Moss, 1989; Flint, 2010), and decision-making accuracy (Day et al., 2009). For example, Day et al. explored the effects of musical tempo and task difficulty on multi-attribute decision-making by using eye-tracking. They concluded that with the same decision time, participants made decisions more accurately when listening to faster rather than slower music. In addition, faster music improved accuracy in more difficult decision-making but not in easier decision-making tasks.

However, in the present study, the participants were prompted to be careful to avoid incorrect answers; therefore, they may have slowed their responses and ignored the fast beat of the background music to ensure that their answers were correct. In activities not requiring complex cognitive effort, following the beat may be easier. Moreover, the nonsignificant results for accuracy in two of the tasks suggested that the participants in the FT condition were as careful not to select the wrong option as those in the DT and NM conditions. Therefore, we can conclude that in cognitive tasks, especially tasks that require more careful consideration, fast music does not have a significant effect on cognitive speed.

The results also revealed that slow music significantly decreases processing speed and, as a result, negatively affects cognitive performance. This result is consistent with the findings of the Mayfield and Moss (1989), in which “heartbeat,” or slow, music caused people to slow down and resulted in poorer performance in this test. This adds evidence confirming that slow music tends to reduce thinking speed. Hence, slow music should not be played in the background when individuals must perform cognitive tasks that have time limits and must be completed quickly.

Another interesting finding is that in the spatial processing task, the accuracy rate of people under the ST condition was higher than that of those in the other conditions, whereas the accuracy rate did not differ between conditions in the two other tasks (motor processing and visual processing). Therefore, in tasks that require more complex processing, music that is slower and more relaxed could increase carefulness. Although the higher accuracy rate was not significant in this case, it might be beneficial in situations without a time limit.

Our second hypothesis (H2) predicted that arousal and mood while listening to music mediates the relationship between musical tempo and processing speed. This hypothesis was rejected because we observed no significant effect of arousal, valence, or dominance on processing speed. Specifically, musical tempo had a positive influence on arousal level but no influence on valence or dominance. This result is consistent with a previous study that tested the effect of musical tempo and mode on arousal and mood (Pelletier, 2004). In that study, the researcher concluded that the musical tempo exerted effects on arousal but not mood, whereas the mode had effects on mood but not arousal.

Although arousal level was affected by the musical tempo, it did not have a significant effect on processing speed. This could be explained by the Yerkes–Dodson Law (Diamond et al., 2007), which indicates that only optimum arousal level (not too low and not too high) can increase performance. Individuals in a low arousal state tend to have low performance. As arousal increases, attention and interest increase simultaneously, which results in increased performance. Performance can peak when arousal reaches its optimal level. However, when arousal level becomes too high, the performance starts to worsen because the arousal causes stress and impairs attention, working memory, and other cognitive abilities. In the current study, music exposure duration may be insufficient for the participants to be stimulated by the background music, which led to failure to reach the optimal level of stimulation. Therefore, the participants appeared to have little arousal; thus, arousal did not have a mediating effect. Although the increased level of arousal caused by musical tempo did not result in higher processing speed and cognitive performance, it was insufficient to cause impairment in attention and thus reduce processing speed and performance.

### 5.1. Practical implications

The practical implications of the study go beyond the obtained results, given the fact that they refer to predictability in cognitive tasks carried out with music in the background, while the study measured the effects of listening to music of different tempo before performing some tasks. The results of this experiment lead to several suggestions for applying music stimulation before performing cognitive tasks for better productivity in everyday life and work. Although music has been proved to benefit cognitive performance, this experiment on its influence on processing speed, together with many earlier studies, suggests that its application should be carefully considered because its effect varies between different conditions and depends on various aspects. Generally, the influence of music on the speed of performing everyday tasks depends on the tempo of the music as well as the nature of the tasks. For simpler cognitive tasks that do not require deep thinking, listening to fast music before the tasks should be beneficial, whereas a slow tempo may slow natural motor speeds, resulting in less work being done in a limited period. For cognitive tasks that are more complex, listening to slow music prior to the tasks may decrease their completion speed but may be beneficial for accuracy because it slows thinking speed, allowing for more careful consideration.

The findings may inform decisions in choosing music before the cognitive tasks to effectively facilitate work performance without creating side effects. For example, in dealing with administrative affairs, people do not need to expend much effort thinking about tasks, and fast-paced music may be suitable as music. In industrial fields where workers perform relatively simple, routine tasks that require fast movement and some degree of attention, fast-tempo music may help workers stay alert and work faster; slow-tempo music should be avoided because it slows working speed and decreases arousal, resulting in boredom and inattentiveness.

By contrast, in a working environment in which employees such as programmers, accountants, and translators perform tasks that require complex cognitive functioning and high levels of accuracy, listening to slow music prior to works without vocals could be utilized to decrease stress levels and increase precision. This also holds true for learning environments. Learners need to focus on learning often complex concepts and to commit information to memory. Thus, slow music is more suitable than fast prior to they face complex learning activities.

The findings also suggest that when students are studying in school or other environments, teachers can select slow-paced music to help students absorb the knowledge learned with a high degree of accuracy. For example, teachers can play slow music prior to learning activities to enable students to absorb content. In addition, teachers can use slow music to help students absorb content accurately during self-study. If a student is engaged in a dynamic activity or is performing laborious tasks, such as assisting in school cleaning activities, the teacher can play fast-paced music to reduce the student’s completion time.

### 5.2. Limitations and future studies

This experiment had limitations that should be addressed in the future, including the lack of a laboratory environment. The experiment was conducted in a classroom environment that was somewhat noisy, which may have affected the performance of the participants. Furthermore, the lack of facilities prevented the researchers from conducting more sophisticated measurement of arousal level, which limited the ability to obtain a reliable data source for mediator analysis. In addition, the only control variable in the study was frequency of listening music. Future studies should consider music preference, volume, or timbre. Furthermore, we claimed that musical tempo is beneficial depending on the complexity of the task, but maybe could be the “nature.” Cognitive processing speed was divided into three categories: visual, verbal, and motor. Opportunities for participants to engage in intelligence-related information processing activities are limited; the tasks were unrelated to the complexity of the participants, and the complexity of these tasks for the participants may also have affected their choice response time. Therefore, future studies are suggested to explore matching the complexity of tasks to the participants.

In future studies, researchers should conduct additional analysis on the mediators and moderators of the relationship between musical tempo and processing speed. Additionally, different tasks evaluating cognitive functions such as reading, memory, and concentration should be adopted to observe differences in the effect between task types. In addition, more detailed levels of musical tempo can be tested to determine the range of musical tempo that increase processing speed and cognitive performance without causing overly high arousal and distraction.

Then, this study only employed one musical piece for fast tempo or slow tempo, and people usually would think that this is not at all sufficient to draw conclusions about general phenomena related to musical tempo; in addition, this study only investigated the influence of fast tempo or slow tempo on cognitive processing speed, and it was difficult to generalize the result for other music, however, music tempo is a continuous quantity, and it is very possible to have different musical tempos in the same song. In addition, participants’ played an instrument would influence their cognitive processing speed, and the future research will take participants played an instrument into account.

Finally, in this study, participants are divided into three groups of different conditions. However, it is necessary to take data from a single individual in the three conditions because for some people background music can decrease the task performance while the effect is positive on average, thus, it is necessary to be discussed the task performance under different music tempo for a single individual, and even investigates in different fields in the future study.

## Data availability statement

The raw data supporting the conclusions of this article will be made available by the authors, without undue reservation.

## Author contributions

All authors listed have made a substantial, direct, and intellectual contribution to the work and approved it for publication.

## Conflict of interest

The authors declare that the research was conducted in the absence of any commercial or financial relationships that could be construed as a potential conflict of interest.

## Publisher’s note

All claims expressed in this article are solely those of the authors and do not necessarily represent those of their affiliated organizations, or those of the publisher, the editors and the reviewers. Any product that may be evaluated in this article, or claim that may be made by its manufacturer, is not guaranteed or endorsed by the publisher.

## References

[ref1] Anderson S. A. Fuller G. B. (2010). Effect of music on reading comprehension of junior high school students. Sch. Psychol. Q. 25, 178–187. doi: 10.1037/a0021213

[ref2] Avila C. Furnham A. McClelland A. (2012). The influence of distracting familiar vocal music on cognitive performance of introverts and extraverts. Psychol. Music 40, 84–93. doi: 10.1177/0305735611422672

[ref3] Bloor A. J. (2009). The rhythm’s gonna get ya’—background music in primary classrooms and its effect on behaviour and attainment. Emot. Behav. Diffic. 14, 261–274. doi: 10.1080/13632750903303070

[ref4] Braaten E. Willoughby B. (2014). Bright Kids Who Can’t Keep Up: Help Your Child Overcome Slow Processing Speed and Succeed in a Fast-Paced World. New York, NY: The Guilford Press.

[ref001] Bradford D. Atri A. (2014). *Dementia: Comprehensive Principles and Practices* Oxford University Press.

[ref002] Bradley M. M. Lang P. J. (1994). Measuring emotion: the self-assessment manikin and the semantic differential. J. Behav. Ther. Exp. Psychiatry. 25, 49–59. doi: 10.1016/0005-7916(94)90063-9 7962581

[ref5] Brodsky W. (2001). The effects of music tempo on simulated driving performance and vehicular control. Transport. Res. F: Traffic Psychol. Behav. 4, 219–241. doi: 10.1016/S1369-8478(01)00025-0

[ref7] Chie Q. T. Karthigeyan K. K. (2009). The effects of music tempo on memory performance using maintenance rehearsal and imagery. Sunw. Acad. J. 6, 114–132.

[ref8] Cohen J. (1988). Statistical Power Analysis for the Behavioral Sciences. New York, NY: Routledge Academic

[ref9] Day R. F. Lin C. H. Huang W. H. Chuang S. H. (2009). Effects of music tempo and task difficulty on multi-attribute decision-making: an eye-tracking approach. Comput. Hum. Behav. 25, 130–143. doi: 10.1016/j.chb.2008.08.001

[ref004] Dehn M. J. (2011). Working Memory and Academic Learning: Assessment and Intervention John Wiley & Sons.

[ref005] Diamond D. M. Campbell A. M. Park C. R. Halonen J. Zoladz P. R. (2007). The temporal dynamics model of emotional memory processing: a synthesis on the neurobiological basis of stress-induced amnesia, flashbulb and traumatic memories, and the Yerkes-Dodson law. Neural Plast. 60803. doi: 10.1155/2007/60803 PMC1906714 17641736

[ref10] Dillman Carpentier F. R. Potter R. F. (2007). Effects of music on physiological arousal: explorations into tempo and genre. Media Psychol. 10, 339–363. doi: 10.1080/15213260701533045

[ref11] Dosseville F. Laborde S. Scelles N. (2012). Music during lectures: will students learn better? Learn. Individ. Differ. 22, 258–262. doi: 10.1016/j.lindif.2011.10.004

[ref12] Doyle M. Furnham A. (2012). The distracting effects of music on the cognitive test performance of creative and non-creative individuals. Think. Skills Creat. 7, 1–7. doi: 10.1016/j.tsc.2011.09.002

[ref13] Edwards J. D. Wadley V. G. Vance D. E. Wood K. Roenker D. L. Ball K. K. (2005). The impact of speed of processing training on cognitive and everyday performance. Aging Ment. Health 9, 262–271. doi: 10.1080/13607860412331336788, PMID: 16019280

[ref15] Fassbender E. Richards D. Bilgin A. Thompson W. F. Heiden W. (2012). VirSchool: the effect of background music and immersive display systems on memory for facts learned in an educational virtual environment. Comput. Educ. 58, 490–500. doi: 10.1016/j.compedu.2011.09.002

[ref006] Flint M. (2010). The Effects of Music on Physical Productivity. Doctoral dissertation, The Ohio State University.

[ref16] Foran L. M. (2009). Listening to music: helping children regulate their emotions and improve learning in the classroom. Educ. Horiz. 88, 51–58.

[ref18] Furnham A. Trew S. Sneade I. (1999). The distracting effects of vocal and instrumental music on the cognitive test performance of introverts and extraverts. Personal. Individ. Differ. 27, 381–392. doi: 10.1016/S0191-8869(98)00249-9

[ref19] Geethanjali B. Adalarasu K. Jagannath M. Rajasekaran R. (2016). Enhancement of task performance aided by music. Curr. Sci. 111, 1794–1801. doi: 10.18520/cs/v111/i11/1794-1801

[ref20] Graziano A. B. Peterson M. Shaw G. L. (1999). Enhanced learning of proportional math through music training and spatial-temporal training. Neurol. Res. 21, 139–152. doi: 10.1080/01616412.1999.11740910, PMID: 10100200

[ref21] Gundlach R. H. (1932). A quantitative analysis of Indian music. Am. J. Psychol. 44, 133–145. doi: 10.2307/1414960

[ref22] Hallam S. Price J. (1998). Can the use of background music improve the behaviour and academic performance of children with emotional and behavioural difficulties? Br. J. Spec. Educ. 25, 88–91. doi: 10.1111/1467-8527.t01-1-00063

[ref23] Hallam S. Price J. (2003). Research section: can the use of background music improve the behaviour and academic performance of children with emotional and behavioural difficulties? Br. J. Spec. Educ. 25, 88–91. doi: 10.1111/1467-8527.t01-1-00063

[ref24] Hallam S. Price J. Katsarou G. (2002). The effects of background music on primary school pupils’ task performance. Educ. Stud. 28, 111–122. doi: 10.1080/03055690220124551

[ref25] Hayes A. F. (2017). Introduction to Mediation, Moderation, and Conditional Process Analysis: A Regression-Based Approach. New York, NY: Guilford publications.

[ref26] Hetland L. (2000). Learning to make music enhances spatial reasoning. J. Aesthet. Educ. 34, 179–238. doi: 10.2307/3333643

[ref27] Hevner K. (1937). The affective value of pitch and tempo in music. Am. J. Psychol. 49, 621–630. doi: 10.2307/1416385

[ref28] Holbrook M. B. Anand P. (1990). Effects of tempo and situational arousal on the listener’s perceptual and affective responses to music. Psychol. Music 18, 150–162. doi: 10.1177/0305735690182004

[ref29] Hu X. Li F. Kong R. (2019). “Can background music facilitate learning? Preliminary results on Reading comprehension.” in Proceedings of the 9th International Conference on Learning Analytics & Knowledge, 101–105.

[ref30] Huang R. H. Shih Y. N. (2011). Effects of background music on concentration of workers. Work 38, 383–387. doi: 10.3233/WOR-2011-1141, PMID: 21508527

[ref31] Irish M. Cunningham C. J. Walsh J. B. Coakley D. Lawlor B. A. Robertson I. H.. (2006). Investigating the enhancing effect of music on autobiographical memory in mild Alzheimer’s disease. Dement. Geriatr. Cogn. Disord. 22, 108–120. doi: 10.1159/000093487, PMID: 16717466

[ref32] Jaušovec N. Jaušovec K. Gerlič I. (2006). The influence of Mozart’s music on brain activity in the process of learning. Clin. Neurophysiol. 117, 2703–2714. doi: 10.1016/j.clinph.2006.08.010, PMID: 17029951

[ref33] Jing Y. Jing S. Huajian C. Chuangang S. Yan L. (2012). “The gender difference in distraction of background music and noise on the cognitive task performance.” in 2012 8th International Conference on Natural Computation. 584–587.

[ref34] Kallinen K. (2002). Reading news from a pocket computer in a distracting environment: effects of the tempo of background music. Comput. Hum. Behav. 18, 537–551. doi: 10.1016/S0747-5632(02)00005-5

[ref007] Kauranen K. (1999). Human Motor Performance and Physiotherapy: Effect of Strapping, Hot and Cold Pack Treatments and Muscle Strength Training. Doctoral dissertation, University of Oulu.

[ref008] Kotsopoulou A. Hallam S. (2010). The perceived impact of playing music while studying: age and cultural differences. Educ. Stud. 36, 431–440. doi: 10.1080/03055690903424774

[ref35] Kuribayashi R. Nittono H. (2015). Speeding up the tempo of background sounds accelerates the pace of behavior. Psychol. Music 43, 808–817. doi: 10.1177/0305735614543216

[ref36] Landay K. Harms P. D. (2019). Whistle while you work? A review of the effects of music in the workplace. Hum. Resour. Manag. Rev. 29, 371–385. doi: 10.1016/j.hrmr.2018.06.003

[ref37] Legutko R. S. Trissler T. T. (2012). The effects of background music on learning disabled elementary school students’ performance in writing. Curr. Issues Educ. 15, 1–10.

[ref38] Lehmann J. A. M. Hamm V. Seufert T. (2019). The influence of background music on learners with varying extraversion: seductive detail or beneficial effect? Appl. Cogn. Psychol. 33, 85–94. doi: 10.1002/acp.3509

[ref39] Lehmann J. A. Seufert T. (2017). The influence of background music on learning in the light of different theoretical perspectives and the role of working memory capacity. Front. Psychol. 8:1902. doi: 10.3389/fpsyg.2017.01902, PMID: 29163283 PMC5671572

[ref40] Macan T. H. Shahani C. Dipboye R. L. Phillips A. P. (1990). College students’ time management: correlations with academic performance and stress. J. Educ. Psychol. 82, 760–768. doi: 10.1037/0022-0663.82.4.760

[ref41] Mathiesen S. L. Mielby L. A. Byrne D. V. Wang Q. J. (2020). Music to eat by: a systematic investigation of the relative importance of tempo and articulation on eating time. Appetite 155:104801. doi: 10.1016/j.appet.2020.104801, PMID: 32682852

[ref42] Mayfield C. Moss S. (1989). Effect of music tempo on task performance. Psychol. Rep. 65, 1283–1290. doi: 10.2466/pr0.1989.65.3f.1283 2623126

[ref44] Morley I. (2013). The Prehistory of Music: Human Evolution, Archaeology, and the Origins of Musicality. UK: Oxford University Press.

[ref009] Mozart W. A. (1985). Sonata for two pianos in D major, K 448 (K. 3375a) [Recorded by M. Perahia & R. Lupu]. On Music for Piano, Four Hands.

[ref45] Nouchi R. Taki Y. Takeuchi H. Hashizume H. Akitsuki Y. Shigemune Y.. (2012). Brain training game improves executive functions and processing speed in the elderly: a randomized controlled trial. PLoS One 7:e29676. doi: 10.1371/journal.pone.0029676, PMID: 22253758 PMC3256163

[ref46] Orini M. Bailón R. Enk R. Koelsch S. Mainardi L. Laguna P. (2010). A method for continuously assessing the autonomic response to music-induced emotions through HRV analysis. Med. Biol. Eng. Comput. 48, 423–433. doi: 10.1007/s11517-010-0592-3, PMID: 20300873

[ref010] Pelletier C. L. (2004). The effect of music on decreasing arousal due to stress: a meta-analysis. J. Music Ther. 41, 192–214. doi: 10.1093/jmt/41.3.192 15327345

[ref011] Posner M. I. Mitchell R. F. (1967). Chronometric analysis of classification. Psychol. Rev. 74, 392–409. doi: 10.1037/h0024913 6076470

[ref47] Rainey D. W. Larsen J. D. (2002). The effect of familiar melodies on initial learning and long-term memory for unconnected text. Music. Percept. 20, 173–186. doi: 10.1525/mp.2002.20.2.173

[ref49] Rigg M. G. (1940). Speed as a determiner of musical mood. J. Exp. Psychol. 27, 566–571. doi: 10.1037/h0058652

[ref50] Ritter S. M. Ferguson S. (2017). Happy creativity: listening to happy music facilitates divergent thinking. PLoS One 12:e0182210. doi: 10.1371/journal.pone.0182210, PMID: 28877176 PMC5587106

[ref51] Roballey T. C. McGreevy C. Rongo R. R. Schwantes M. L. Steger P. J. Wininger M. A.. (1985). The effect of music on eating behavior. Bull. Psychon. Soc. 23, 221–222. doi: 10.3758/BF03329832

[ref012] Ruff R. M. Parker S. B. (1993). Gender- and age-specific changes in motor speed and eye-hand coordination in adults: normative values for the Finger Tapping and Grooved Pegboard Tests. Percept. Mot. Skills. 76, 1219–1230. doi: 10.2466/pms.1993.76.3c.1219 8337069

[ref52] Sacks O. (2007). Musicophilia. New York, NY: Vintage Books

[ref53] Salamé P. Baddeley A. (1989). Effects of background music on phonological short-term memory. Q. J. Exp. Psychol. Sec. A. 41, 107–122. doi: 10.1080/14640748908402355

[ref54] Sandroff B. M. Motl R. W. (2012). Fitness and cognitive processing speed in persons with multiple sclerosis: a cross-sectional investigation. J. Clin. Exp. Neuropsychol. 34, 1041–1052. doi: 10.1080/13803395.2012.715144, PMID: 22905722

[ref013] Savan A. (1999). The effect of background music on learning. Psychol. Music. 27, 138–146. doi: 10.1177/030573569927

[ref55] Scherer K. R. Oshinsky J. S. (1977). Cue utilization in emotion attribution from auditory stimuli. Motiv. Emot. 1, 331–346. doi: 10.1007/BF00992539

[ref56] Schwartz R. W. Ayres K. M. Douglas K. H. (2017). Effects of music on task performance, engagement, and behavior: a literature review. Psychol. Music 45, 611–627. doi: 10.1177/0305735617691118

[ref57] Smith C. A. Morris L. W. (1977). Differential effects of stimulative and sedative music on anxiety, concentration, and performance. Psychol. Rep. 41, 1047–1053. doi: 10.2466/pr0.1977.41.3f.1047, PMID: 601132

[ref58] Spence C. (2011). Crossmodal correspondences: A tutorial review. Atten. Percept. Psychophysiol. 73, 971–995. doi: 10.3758/s13414-010-0073-7, PMID: 21264748

[ref59] Spence C. Piqueras-Fiszman B. (2014). The Perfect Meal: The Multisensory Science of Food and Dining. Oxford, UK: Wiley-Blackwell

[ref60] Spence C. Reinoso-Carvalho F. Velasco C. Wang Q. J. (2019). Extrinsic auditory contributions to food perception & consumer behaviour: an interdisciplinary review. Multisens. Res. 32, 275–318. doi: 10.1163/22134808-20191403, PMID: 31059484

[ref61] Styhre A. (2013). Sound, silence, music: organizing audible work settings. Cult. Organ. 19, 22–41. doi: 10.1080/14759551.2011.634197

[ref62] Sweet L. H. (2011). “Information Processing Speed,” in Encyclopedia of Clinical Neuropsychology. eds. Kreutzer J. S. DeLuca J. Caplan B. (New York, NY: Springer), 1317–1318.

[ref63] Thompson W. F. Schellenberg E. G. Letnic A. K. (2012). Fast and loud background music disrupts reading comprehension. Psychol. Music 40, 700–708. doi: 10.1177/0305735611400173

[ref014] Tombaugh T. N. Berrigan L. I. Walker L. A. S. Freedman M. S. (2010). The Computerized Test of Information Processing (CTIP) offers an alternative to the PASAT for assessing cognitive processing speed in individuals with multiple sclerosis. Cogn. Behav. Neurol. 23, 192–198. doi: 10.1097/WNN.0b013e3181cc8bd4 20829669

[ref64] Walker L. A. S. Cheng A. Berard J. Berrigan L. I. Rees L. M. Freedman M. S. (2012). Tests of information processing speed: what do people with multiple sclerosis think about them? Int. J. MS Care 14, 92–99. doi: 10.7224/1537-2073-14.2.92, PMID: 24453739 PMC3883004

[ref65] Watson K. B. (1942). The nature and measurement of musical meanings. Psychol. Monogr. Gen. Appl. 54, 1–43. doi: 10.1037/h0093496

[ref015] Wolfe J. M. (2018). Stevens’ Handbook of Experimental Psychology and Cognitive Neuroscience, Viusal Search (Vol. 2). New York, NY: John Wiley & Sons.

[ref67] Yerkes R. M. Dodson J. D. (1908). The relation of strength of stimulus to rapidity of habit-formation. J. Comp. Neurol. Psychol. 18, 459–482. doi: 10.1002/cne.920180503

[ref68] Zhou Y. Siu A. F. (2015). Motivational intensity modulates the effects of positive emotions on set shifting after controlling physiological arousal. Scand. J. Psychol. 56, 613–621. doi: 10.1111/sjop.12247, PMID: 26453484

